# Editorial

**Published:** 1868-04

**Authors:** 


### Editorial.

Medical College Commencements in Chicago.—Several weeks since, Rush Medical College closed its regular annual college term, by conferring the degree of M.D. upon 120 candidates for graduation. The degrees were conferred by the President of the College, Prof. J. V. Z. Blaney, and the regular valedictory address was delivered by Prof. R. L. Rea. The exact number of students in attendance during the past winter we have not seen stated, but think it was not far from 275.

The Chicago Medical College celebrated its ninth annual commencement on the 3d of March. The afternoon of the preceding day had been spent in the reading of theses by their authors, and the *public* examination of the candidates for graduation. At the commencement proper, the President of the Faculty first delivered the certificates to such members of the Junior Class as had passed satisfactory examinations in the Junior Series of branches, accompanying the same by a brief exhortation in relation to the further prosecution of their studies. Certificates were next given by the Professor of Clinical Surgery, to those members of the class who had served a specified time as dressers in the surgical wards of Mercy Hospital.

Dr. J. S. Hildreth, Surgeon to the Eye and Ear Department of the County Hospital, conferred special certificates on seven of the candidates for graduation, who had not only attended his clinics in the Hospital, but had also passed a satisfactory examination by him, on the subject of Ophthalmology. He accompanied the certificates by a short and very appropriate address.

The Committee of the Faculty, appointed for that purpose, awarded the prize for the best thesis to Nicholas Senn, and for the second best to D. W. Nolan.

The President then conferred the degree of Doctor of Medicine on 45 regular candidates for graduation, and 5 *ad-eundum* and honorary degrees on practitioners who had been properly recommended for that purpose. The delivery of the diplomas was accompanied by a brief and impressive charge to the graduates, concerning the nature and importance of the duties and responsibilities they assumed in entering upon the active practice of the profession of medicine.

E. S. Cleveland, in behalf of the class, responded to the charge of the President in a very neat and appropriate address.

The exercises were closed by a valedictory address by Prof. E. Andrews, on the Recreations of the Physician; a subject which was treated in a most interesting and instructive manner.

It will be seen by this brief statement, that the Chicago Medical College, though organized, from its first establishment, on the basis of a long lecture-term; consecutive order of studies (the branches being divided into junior and senior series), annual examinations; and hospital clinical instruction as an integral part; with higher lecture-fees than any other school in this region; has advanced with steadiness and rapidity to a high degree of prosperity.

Indeed, the City of Chicago has not advanced more rapidly in her commercial or material interests than she has in the growth of her medical schools. Ten years since, she had but one medical college and one hospital. The number of medical students in attendance was about 130, and the number of degrees conferred about 40. Now we have 2 colleges, 4 hospitals, 400 students, and 170 graduates, making her rank next to New York and Philadelphia, as a great centre of medical college and hospital instruction.

Alumni Association of the Chicago Medical College.—The first anniversary meeting of this association was held in the College Hall, immediately after the close of the commencement exercises. The President of the Association, Professor Jewell, delivered an address on the subject of Medical Education. He advocated the immediate adoption of all the recommendations of the Cincinnati Convention, and presented the whole subject in a style so clear and interesting as to fix the attention and elicit the approval of his audience.

---

Illinois State Medical Society.—The next Annual Meeting of the Illinois State Medical Society will be held in the City of *Quincy*, commencing on the *Third Tuesday* in May, 1868. It is very desirable that the profession throughout the State should be well represented.

N. S. DAVIS,

*March 26th, 1868.* *Permanent Sec'y.*

---

### AMERICAN MEDICAL ASSOCIATION.

---

Office of Permanent Secretary, Wm. B. Atkinson, M.D.,
*S.-W. Cor. Broad and Pine Sts., Phila.*

The Nineteenth Annual Meeting of the American Medical Association will be held in Washington, on Tuesday, May 5th, 1868, at 11 o'clock A.M.

The following Committees are expected to report:

On Ophthalmology, Dr. Jos. S. Hildreth, Illinois, Chairman.

On Cultivation of the Cinchona Tree, Dr. J. M. Toner, D.C., Chairman.

On Surgical Diseases of Women, Dr. Theophilus Parvin, Ind., Chairman.

On Rank of Medical Men in the Navy, Dr. N. S. Davis, Ill., Chairman.

On Insanity, Dr. C. A. Lee, N.Y., Chairman.

On American Medical Necrology, Dr. C. C. Cox, Md., Chair'n.

On Leakage of Gas-Pipes, Dr. J. C. Draper, N.Y., Chairman.

On Medical Ethics, ——— Chairman.

On Plan of Organization, Dr. C. C. Cox, M.D., Chairman.

On Provision for the Insane, Dr. C. A. Lee, N.Y., Chairman.

On the Climatology and Epidemics of Maine, Dr. J. C. Weston; of New Hampshire, Dr. P. A. Stackpole; Vermont, Dr.

Henry Janes; Massachusetts, Dr. Alfred C. Garratt; Rhode Island, Dr. C. W. Parsons; Connecticut, Dr. E. K. Hunt; New York, Dr. W. F. Thoms; New Jersey, Dr. Ezra M. Hunt; Pennsylvania, Dr. D. F. Condie; Maryland, Dr. O. S. Mahon; Georgia, Dr. Juriah Harriss; Missouri, Dr. Geo. Engelman; Alabama, Dr. R. Miller; Texas, Dr. T. J. Heard; Illinois, Dr. R. C. Hamil; Indiana, Dr. J. F. Hibberd; District of Columbia, Dr. T. Antisell; Iowa, Dr. J. W. H. Baber; Michigan, Dr. Ab'm Sager; Ohio, Dr. W. Russell; California, Dr. F. W. Hatch; Tennessee, Dr. Joseph Jones; W. Virginia, Dr. E. A. Hildreth; Minnesota, Dr. Sam'l Willey.

On Clinical Thermometry in Diphtheria, Dr. Jos. G. Richardson, N.Y., Chairman.

On the Treatment of Disease by Atomized Substance, Dr. A. G. Field, Iowa, Chairman.

On the Ligation of Arteries, Dr. Benj. Howard, N.Y., Ch'n.

On the Treatment of Club-Foot without Tenotomy, Dr. L. A. Sayre, N.Y., Chairman.

On the Radical Cure of Hernia, Dr. G. C. Blackman, Ohio, Chairman.

On Operations for Harelip, Dr. Hammer, Mo., Chairman.

On Errors of Diagnosis in Abdominal Tumors, Dr. G. C. E. Weber, Ohio, Chairman.

On Prize Essays, Dr. Chas. Woodward, Ohio, Chairman.

On Medical Education, Dr. A. B. Palmer, Mich., Chairman.

On Medical Literature, Dr. Geo. Mendenhall, Ohio, Chairman.

Secretaries of all medical organizations are requested to forward lists of their delegates, as soon as elected, to the Permanent Secretary.

W. B. ATKINSON.

---

JUST PUBLISHED.

---

A Complete List of the Muscles of the Human Body, by WILLIAM LITTLE, M.D., Chicago. A Chart, size 24x38, giving the name, origin, insertion, and use of all the muscles in the Human Body. Price 50 cents; mounted on rollers, $1.50.

Also, the Cranial Nerves, Twelve Pairs, giving the name, origin, foramen of exit, distribution, and function. Price 20 cents. Address, WILLIAM LITTLE, M.D.,

*220 Indiana St., Chicago.*

### Mortality Report for the Month of February:—

### CAUSES OF DEATH.

| | | | | | |
|---|---|---|---|---|---|
| Accidents | 6 | Croup | 12 | Measles, following the | 3 |
| Apoplexy | 6 | Debility | 12 | Meningitis | 5 |
| Bowels, inflammation | 2 | Diphtheria | 6 | Paralysis | 2 |
| Brain, congestion of | 6 | Dropsy | 3 | Peritonitis | 4 |
| Brain, disease of | 2 | Dysentery | 4 | Phthisis pulmonalisis | 41 |
| Brain, inflammation | 5 | Encephalites | 2 | Pneumonia | 52 |
| Birth, premature | 9 | Fever, puerperal | 5 | Pneumonia, typhoid | 2 |
| " still | 36 | Fever, scarlet | 5 | Scrofula | 2 |
| " tedious | 1 | Fever, typhoid | 7 | Small-pox | 33 |
| Bronchitis | 7 | Heart, disease of | 7 | Tabes mesenterica | 9 |
| Bronchitis, capillary | 4 | Hydrocephalus | 8 | Teething | 5 |
| Cerebritis | 2 | Inanition | 8 | Varioloid | 2 |
| Childbirth | 3 | Lungs, hemorrhage of | 2 | Unknown | 2 |
| Cholera infantum | 2 | Lungs, congestion of | 5 | | |
| Convulsions | 41 | Measles | 24 | Total | 423 |

Deaths in Feb'y, 1868, 423 | Deaths in Feb'y, 1867, 265 | Decrease, 158
Deaths in January, 1868, 438 | Increase, 15

### AGES.

| | | | | | |
|---|---|---|---|---|---|
| Under 5 | 254 | 40 to 50 | 22 | 90 to 100 | 0 |
| 5 to 10 | 25 | 50 to 60 | 3 | 100 to 110 | 0 |
| 10 to 20 | 15 | 60 to 70 | 12 | Unknown | 0 |
| 20 to 30 | 42 | 70 to 80 | 8 | | |
| 30 to 40 | 38 | 80 to 90 | 4 | Total | 423 |

| | | | | | |
|---|---|---|---|---|---|
| Males, | 221 | Females, | 202 | Total, | 423 |
| Single, | 335 | Married, | 88 | Total, | 423 |
| White, | 420 | Colored, | 3 | Total, | 423 |

### NATIVITY.

| | | | | | |
|---|---|---|---|---|---|
| Chicago | 198 | Germany | 41 | Russia | 0 |
| Other parts U. S. | 104 | Holland | 9 | Scotland | 4 |
| Australia | 0 | Ireland | 30 | Sandwich Islands | 0 |
| Belgium | 0 | Isle of Man | 1 | Sweden | 10 |
| Bohemia | 1 | Norway | 10 | Switzerland | 0 |
| Canada | 3 | Nova Scotia | 1 | Wales | 0 |
| Denmark | 0 | Prussia | 5 | Unknown | 0 |
| England | 7 | Poland | 0 | | |
| France | 1 | Prince Edward Isla. | 1 | Total | 423 |

### MORTALITY BY WARDS FOR THE MONTH.

| Ward. | Mortality. | Pop. in 1866. | One death in | Ward. | Mortality. | Pop. in 1866. | One death in |
|---|---|---|---|---|---|---|---|
| 1 | 10 | 9,668 | 1,208 1-2 | 14 | 25 | 12,108 | 448 12-27 |
| 2 | 21 | 12,985 | 811 9-16 | 15 | 47 | 15,766 | 404 1-4 |
| 3 | 18 | 15,738 | 655 9-12 | 16 | 35 | 14,912 | 573 7-13 |
| 4 | 19 | 10,884 | 380 1-8 | County hosp. | 10 | | |
| 5 | 30 | 9,610 | 536 1-9 | HosWom.Chi. | 3 | | |
| 6 | 18 | 10,680 | 391 7-9 | Soldier's Ho. | 1 | | |
| 7 | 43 | 18,755 | 538 5-35 | Home of the Friendl's, | 2 | | |
| 8 | 17 | 10,429 | 453 10-23 | Marine hos., | 0 | | |
| 9 | 20 | 13,940 | 422 14-33 | Mercy hos., | 1 | | |
| 10 | 17 | 11,416 | 634 2-9 | St.Luke's hos. | 0 | | |
| 11 | 32 | 12,924 | 359 | St.Jose. asyl. | 6 | | |
| 12 | 28 | 12,695 | 396 2-3 | | | | |
| 13 | 16 | 8,188 | 629 11-13 | Lake Hosp., | 4 | | |

Total, 423

DEATHS BY SMALL-POX.

For the Month of February, 1868.

| | | | |
|---|---|---|---|
| 1st Ward | 2 | 11th Ward | 2 |
| 3d Ward | 1 | 12th Ward | 1 |
| 4th Ward | 1 | 15th Ward | 4 |
| 5th Ward | 4 | Lake Hospital | 4 |
| 6th Ward | 1 | | |
| 7th Ward | 6 | Total, | 33 |
| 8th Ward | 2 | | |
| 9th Ward | 4 | January, 1868, | 27 |
| 10th Ward | 1 | Varioloid, 2d Ward, | 2 |

CITY PHYSICIAN'S REPORT.

It appears from the Report of the City Physician, that

| | |
|---|---|
| The whole number of patients remaining in Hospital, February 1, was | 45 |
| Admitted during the month, | 68 |
| Total, | 113 |

Discharged cured, 61; Died, 6; Remaining at Hospital, 46;

The complaints of those who were discharged cured were:

Variola confluens, 20; Variola coherens, 15; Variola discreta, 21; Rubeola, 4; Scarlatina, 1.

Five persons died of variola confluens and one of phthisis teberculosis. Of the above five, three had never been vaccinated, and the other two had been vaccinated, but it had not taken.

The diseases of those now in the hospital are as follows:—Variola confluens, 18; Variola coherens, 12; Variola discreta, 16.

---

MONEY RECEIPTS TO MARCH 27TH.—Drs. S. P. Breed, $3; A. W. Gray, 3; Geo. Fredigke, 3; J. F. Kelsey, 3; D. B. Trimble, 3; John P. Seebold, 3; Jas. M. Barlow, 3; W. A. Fox, 3; G. L. Henderson, 3; A. H. DePuy, 3; Asher Goslin, 3; Carter & Frazey, 3; R. C. Moore, 6; H. A. Chase, 3; J. B. Cloud, 3; W. W. Duncan, 3; John D. Wood, 3; W. H. Lyford, 3; J. H. Reynolds, 3; C. G. Reim, 3; A. A. Dunn, 3; G. Bradley, 1.50; John Walker, 3; F. B. Etheridge, 6.

Average Duration of Life in Italy.—The director of the Italian Life Assurance Society, M. W. Rey, has published some interesting statistics showing the average duration of life in Italy as compared with that in other countries, from which it appears that the mortality of Italians is exceptionally great. He shows that in Italy, 22½ per cent. of the infant population die yearly, and that, even in the healthiest districts, the average duration of life is 33.43 years only, while in France it is 38.33, at Geneva, 42.02, and in England, 39.31. The number of births, too, is relatively much smaller in Italy than in England and France.

---

Lamentable Death from Chloroform.—A young lady, the wife of a lieutenant-colonel in the Confederate army, died recently at Gallatin under very painful circumstances. Being near confinement, and suffering during the night with great pain, she inhaled some chloroform on her own account. She laid the handkerchief upon her chin and fell asleep. In the morning she was found dead. This circumstance goes to prove how dangerous a plaything chloroform is.

